# ﻿Notes on *Carex* (Cyperaceae) from China (IX): three new species of section Mitratae s.l.

**DOI:** 10.3897/phytokeys.225.101410

**Published:** 2023-04-27

**Authors:** Yi-Fei Lu, Zhao-Cen Lu, Yu-Hao Duan, Kun Zhang, Xiao-Feng Jin

**Affiliations:** 1 Zhejiang Provincial Key Laboratory of Forest Aromatic Plants-based Healthcare Functions and School of Forestry and Biotechnology, Zhejiang A&F University, Hangzhou, Zhejiang 311300, China Zhejiang A&F University Hangzhou China; 2 Guangxi Key Laboratory of Plant Conservation and Restoration Ecology in Karst Terrain, Guangxi Institute of Botany, Guangxi Zhuang Autonomous Region and Chinese Academy of Sciences, Guilin, 541006, Guangxi, China Guangxi Institute of Botany, Guangxi Zhuang Autonomous Region and Chinese Academy of Sciences Guilin China

**Keywords:** *Carex* sect. *Mitratae*, China, new species, sect. *Lageniformes*

## Abstract

Carexsect.Mitratae s.l. was established by Kükenthal in 1909 and can be distinguished from the closely related sections in having nutlets frequently discoid-annulate at the apex and a persistent style base. Based on field surveys and specimen examination, three new species of sect. Mitratae are described and illustrated here. *Carexfatsuaniana* was collected from Yunnan and differs from *C.truncatigluma* in having the utricles nearly glabrous, the nutlets with a ca. 0.5 mm long beak at the apex, the staminate spikes cylindrical, 5–7.5 cm long, 4–5 mm wide, and the pistillate glumes acuminate at the apex. *Carexdamingshanica* was collected from Guangxi and differs from *C.breviscapa* and *C.rhynchachaenium* in having 3 or 4 spikes, the lateral spikes cylindrical, the pistillate glumes, utricles and nutlets all shorter than in the other two species. *Carexradicalispicula* was collected from Sichuan and differs from *C.truncatirostris* in having the staminate spikes clavate, 1.5–2 mm wide, the pistillate glumes pale yellow-white, 3–3.2 mm long, acuminate or short-awned at the apex, and the nutlets with 3 angles shallowly constricted at the middle.

## ﻿Introduction

*Carex* L. (Cyperaceae), a morphological diverse genus with about 2,000 species, is one of the largest genera of angiosperms and is distributed on all continents except Antarctica (Reznicek 1990; Govaerts et al. 2021; Pender et al. 2021). The main characters of this genus that distinguish it from the other genera in the Cyperaceae are flowers unisexual, the female ones contained within a prophyllar structure called a perigynium, which is referred to as a utricle when its margins are fused and closed (Dai et al. 2010; Jiménez-Mejías et al. 2016). Following an increasing number of samples and molecular markers, the systematic framework of *Carex* has become more robust, six strongly supported distinct main lineages were detected, viz. the *Siderostictae*, *Schoenoxiphium*, *Unispicate*, *Uncinia*, *Vignea* and core *Carex* clades (Villaverde et al. 2020; Roalson et al. 2021). A large number of new species of the core *Carex* clade were described during, or soon after, the preparation of the “Flora of China” (Dai et al. 2010; Lu and Jin 2022; Lu et al. 2022).

Carexsect.Mitratae Kük. s.l. (Kükenthal 1909), containing 80+ species, was traditionally divided into three sections: *Cryptostachyae* Franch., *Lageniformes* (Ohwi) Nelmes and *Mitratae* s.s. These are mainly distributed from E and SE Asia to Australia and New Zealand, with a few species reaching Europe, as well as into western and northern Asia (Akiyama 1955; Dai et al. 2010; Roalson et al. 2021). Recent phylogenetic studies revealed the sect. Mitratae s.l. is a polyphyletic group, and five clades which were named as Sect. Cryptostachyae, Tristachya Clade, Truncatigluma Clade, Mitrata Clade and Conica Clade in the core *Carex* clade are recognized (Roalson et al. 2021).

The group, sect. Mitratae s.l., is easily recognized on some morphological characters such as nutlet shape and utricle shape, as well as growth habits. During the field surveys and specimen examination of *Carex*, and during preparation of a taxonomic monograph of sect. Mitratae s.l., three new species were discovered, which are described below.

## ﻿Taxonomic treatment

### ﻿1. Carex fatsuaniana

Taxon classificationPlantaePoalesCyperaceae

X.F.Jin, Y.F.Lu & Z.C.Lu sp. nov.

4CA73E1A-D92F-5730-A530-2029D71B2EAB

urn:lsid:ipni.org:names:77318323-1

Fig. 1A–G

#### Diagnostic description.

This new species is similar to *Carextruncatigluma* C.B.Clarke, but differs in having utricles nearly glabrous, nutlets with a ca. 0.5 mm long beak at apex, staminate spikes cylindrical, 5–7.5 cm long, 4–5 mm wide, and pistillate glumes acuminate at apex.

**Figure 1.**
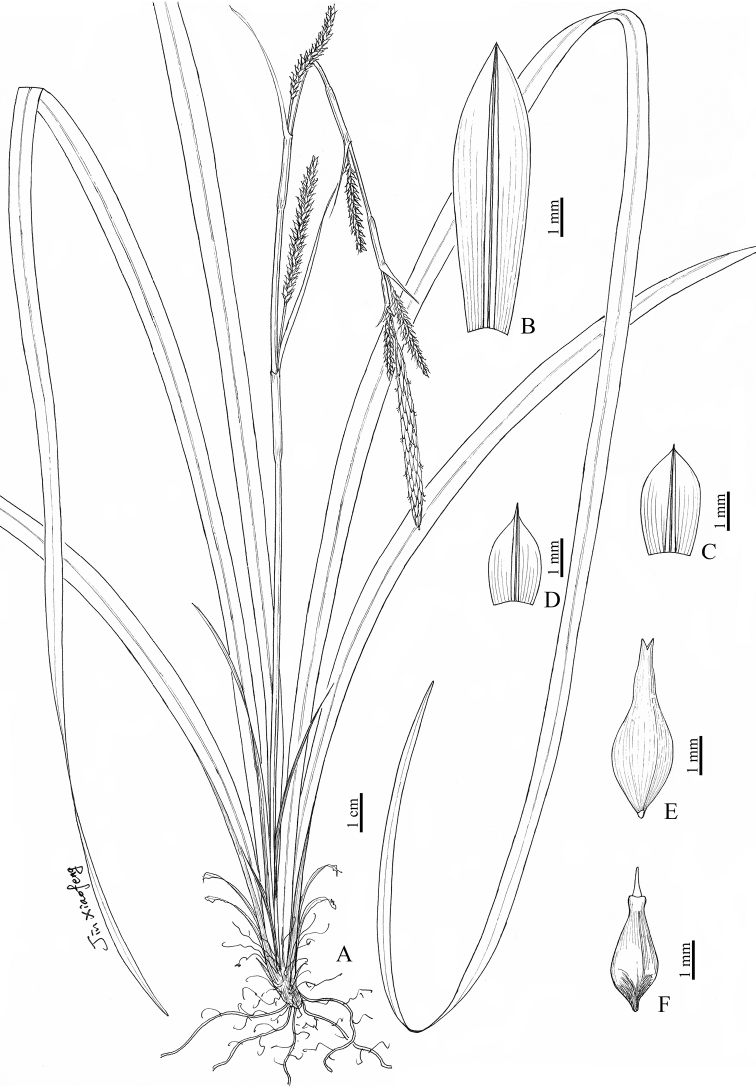
*Carexfatsuaniana* sp. nov. **A** habit **B** staminate glume **C, D** pistillate glume **E** utricle **F** nutlet. (Drawn by Xiao-Feng Jin; based on the holotype: *C. W. Wang 88293* in PE).

#### Type.

China. Yunnan: Foo-ning [Funing County], Ban-loun [Banlun Township], under dense-thickets, alt. 700 m, 10 Apr 1940, *C. W. Wang 88293* (holotype: PE!; isotypes: IBK00181423!, PE!).

#### Description.

Perennial herbs. Rhizomes woody, obliquely ascending. Culms lateral, 45–60 cm tall, trigonous, smooth, base with short leaves and brown fibrous sheaths. Leaves longer than culms, blades flat, leathery, 4.5–9 mm wide, scabrous on upper surfaces and margins. Bracts leaf-like or shortly leaf-like, base with 1–2.5 cm long sheath. Spikes 6; terminal spike staminate, cylindrical, 5–7.5 cm long, 4–5 mm wide, base with 8–20 mm long peduncles; lateral spikes pistillate, cylindrical, 2.5–4.5 cm long, 3–3.5 mm wide, densely flowered, with peduncles exserted from or enclosed in sheaths. Staminate glumes elliptic-lanceolate or oblanceolate, pale brown, 7–7.5 mm long, apex acuminate, with 3-veined yellow-brown costa. Pistillate glumes ovate-elliptic or obovate, pale yellow, 2.5–3 mm long, apex acuminate and mucronate, or acute, with 3-veined yellow-brown costa. Utricles yellow-green, narrowly ellipsoid-ovoid, obtusely trigonous, 4–4.5 mm long, longer than pistillate glumes, membranous, obliquely patent, distinctly thinly veined, nearly glabrous, base gradually cuneate, short-stipitate, apex gradually contracted into a ca. 1 mm long beak, orifice 2-lobed with short teeth. Nutlets tightly enveloped, brown, ovoid, trigonous, 2.5–3 mm long, with 3 sides slightly concave below, base short-stipitate, apex contracted into a ca. 0.5 mm long cylindrical beak; style base slightly thickened; stigmas 3.

#### Etymology.

The specific epithet ‘*fatsuaniana*’ is in honour of Prof. Fa-Tsuan Wang (Fa-Zuan Wang, 1899–1985), the taxonomic founder of Chinese monocots.

#### Phenology.

Flowering and fruiting occur in early April.

#### Conservation status.

Data Deficient (DD). Only four sheets (*C. W. Wang 88293*) of the new species were collected by Chi-Wu Wang in 1940 from the type locality. Adequate information is lacking on its distribution and population status to make a direct or indirect assessment of the risk of extinction (IUCN 2019).

#### Notes.

*Carexfatsuaniana* has nutlets contracted distally into a ca. 0.5 mm long cylindrical beak at the apex, which morphologically belongs to sect. Lageniformes and is similar to *C.truncatigluma* (Dai et al. 2010). In sect. Lageniformes, the species has terminal staminate spikes thinly linear-clavate, whereas those of the new species are cylindrical, 5–7.5 cm long, 4–5 mm wide. The characters distinguishing the new species from *C.truncatigluma* are shown in Table 1.

**Table 1.** Morphological characters distinguishing *Carexfatsuaniana* from *C.truncatigluma*.

Characters	*C.fatsuaniana*	*C.truncatigluma*
1. Staminate spike	Cylindrical, 5–7.5 cm long, 4–5 mm wide	Thinly linear-clavate, 1–2 cm long, 1–2 mm wide
2. Staminate glume	Elliptic-lanceolate or oblanceolate, 7–7.5 mm long, acuminate at apex	Oblong-ovate or ovate, 3–3.5 mm long, obtuse at apex
3. Pistillate glume	Ovate-elliptic or obovate, acuminate and mucronate, or acute at apex	Broadly obovate, obtuse, truncate or emarginate at apex, sometimes short-awned or mucronate.
4. Utricle	Nearly glabrous	Pubescent
5. Nutlet	Beak ca. 0.5 mm long, cylindrical	Beak 0.5–1.5 mm long, thick-cylindrical

Based on the phylogenetic scaffold for the *Carex* classification (Roalson et al. 2021), the sampled species in sect. Lageniformes were arranged in two clades: *Carexbreviscapa* and *C.longicolla* in Tristachya clade, and *Carextruncatigluma* in Truncatigluma clade, but *C.densipilosa* was placed in the uncertain group. The new species, *Carexfatsuaniana*, is mostly closed to *C.truncatigluma* in morphology, so it’s temporarily placed in the Truncatigluma clade.

### ﻿2. Carex damingshanica

Taxon classificationPlantaePoalesCyperaceae

Z.C.Lu & X.F.Jin sp. nov.

B0CBAA34-DEC0-5D1A-8123-F332ED95CD03

urn:lsid:ipni.org:names:77318324-1

Figs 2A–G , 3A–H

#### Diagnostic description.

This new species is similar to *Carexbreviscapa* C.B.Clarke and *C.rhynchachaenium* C.B.Clarke in having spikes in a short racemose and culms much shorter than leaves, but differs from these two relatives in having spikes 3 or 4, lateral spikes cylindrical, shorter, 4–11 mm long, pistillate glumes (1–1.2 mm long), utricles (2.5–3 mm long) and nutlets (1.5–1.9 mm long) all shorter than in related species.

**Figure 2.**
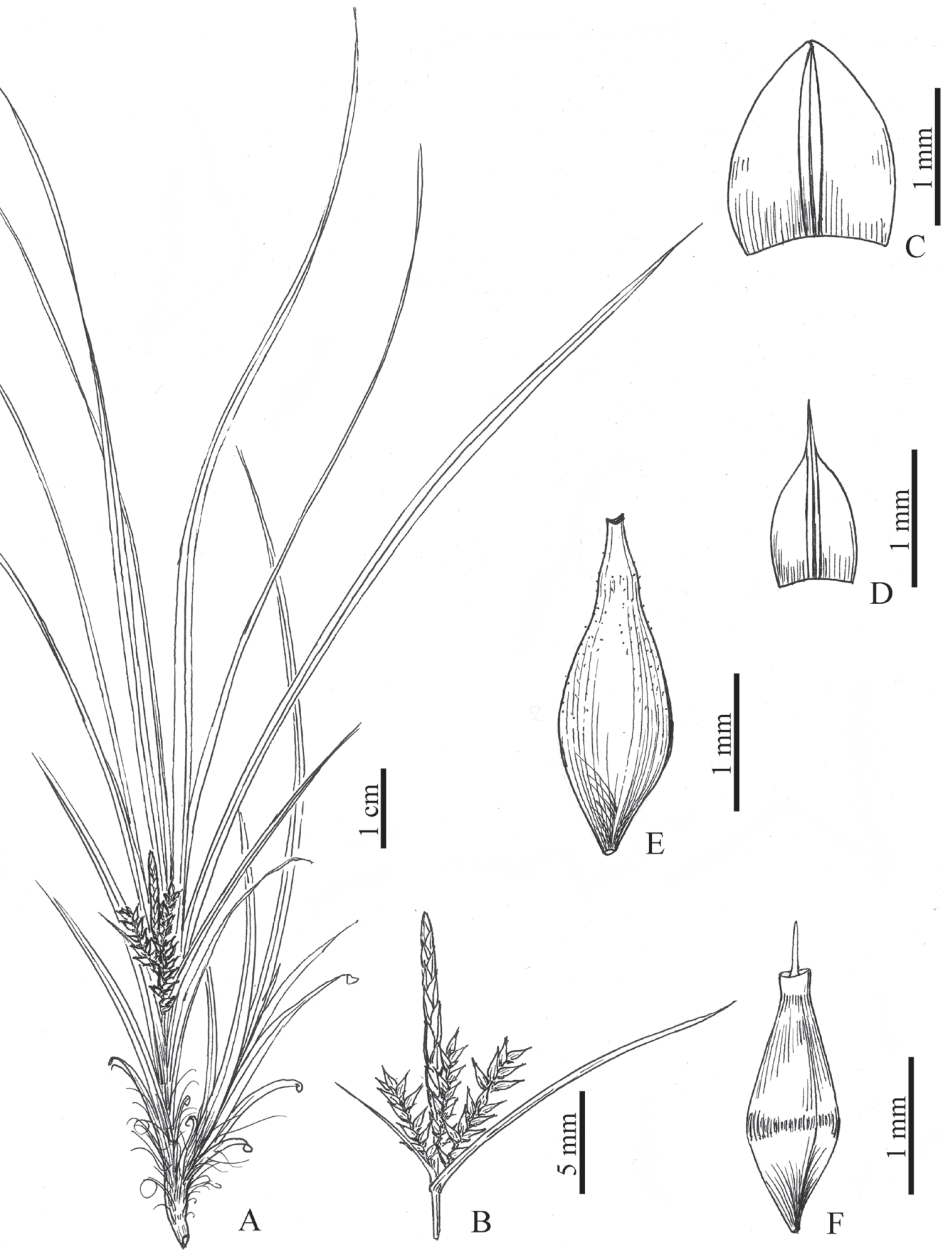
*Carexdamingshanica* sp. nov. **A** habit **B** upper part of inflorescence **C** staminate glume **D** pistillate glume **E** utricle **F** nutlet. (Drawn by Xiao-Feng Jin; based on the holotype: *P. Yang et al. 450125200526074LY* in ZM).

#### Type.

China. Guangxi: Nanning City, Shanglin County, Dafeng Town, Shuiyuan Village, Damingshan, 23°24'53.29"N, 108°31'45.16"E, under broad-leaved forest, alt. 423 m, 26 May 2020, *P. Yang et al. 450125200526074LY* (holotype: ZM!; isotypes: IBK00445399!, IBK00445400!, ZJFC!, ZM!).

**Figure 3.**
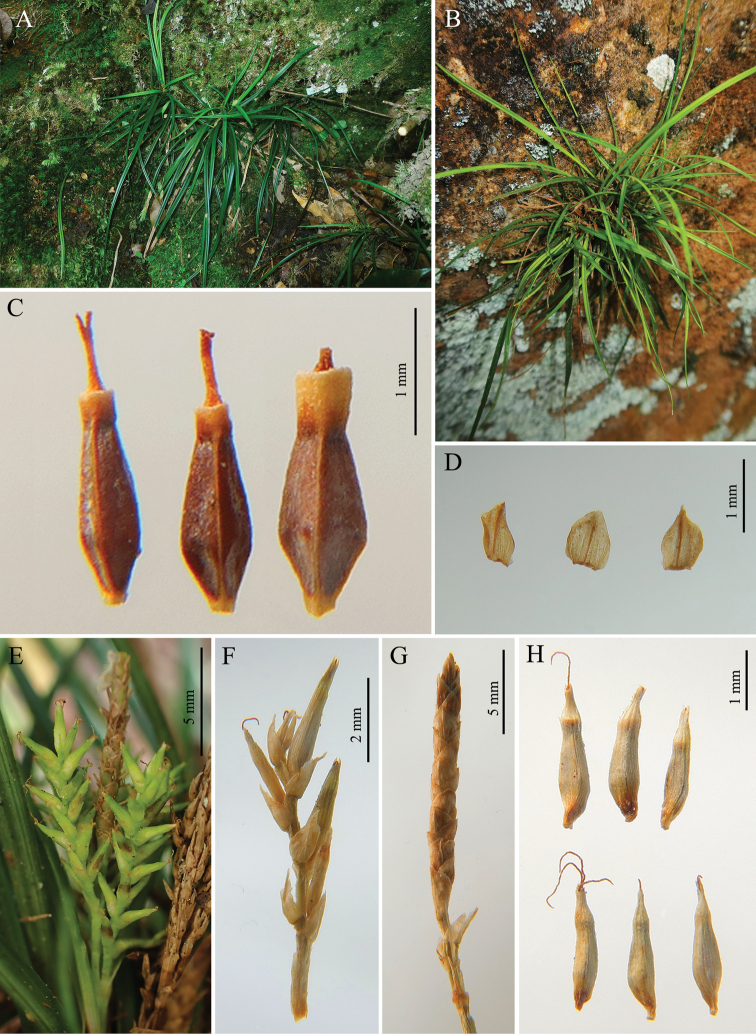
*Carexdamingshanica* sp. nov. **A, B** habit **C** nutlets **D** pistillate glume **E** inflorescence **F** pistillate spike **G** staminate spike **H** utricle. (**C, D, F–H** from type material).

#### Description.

Perennial herbs. Rhizomes short, woody, stiff. Culms central, loosely turfed, 2.5–11 cm tall, trigonous, base with brown fibrous sheaths. Leaves much longer than culms, blades 1.5–3.5 mm wide, flat, leathery, scabrous on upper part and margins. Lowermost bract leaf-like, longer than inflorescence, base with 1–2 mm long sheath or sheathless, others setaceous, shorter than inflorescence, sheathless. Spikes 3 or 4, aggregated; terminal spike staminate, narrowly linear-cylindrical, 6–20 mm long, 1–1.5 mm wide, base with 1–3 mm long peduncles; lateral spikes pistillate, cylindrical, 4–13 mm long, 2.5–3 mm wide, loosely 9–14-flowered, with peduncles slightly exserted from sheaths. Staminate glumes broadly ovate, pale yellow-brown, ca. 1.5 mm long, apex obtuse, with 3-veined yellow costa. Pistillate glumes ovate, pale yellow, 1–1.2 mm long, apex acuminate, with 3-veined yellow costa. Utricles yellow-green, narrowly fusiform, obtusely trigonous, 2.5–3 mm long, longer than pistillate glumes, membranous, obliquely patent, distinctly thinly veined, sparsely pubescent, base gradually cuneate, short-stipitate, apex gradually contracted into a ca. 0.5 mm long beak, orifice 2-lobed with minute teeth. Nutlets tightly enveloped, brown, narrowly ovoid, trigonous, 1.5–1.9 mm long, with 3 sides slightly concave above and below, base with a ca. 0.2 mm long stipe, apex contracted into a 0.2–0.4 mm long cylindrical beak, truncate and shallowly concave at top; style base slightly thickened; stigmas 3.

#### Etymology.

The specific epithet ‘*damingshanica*’ refers to the type locality of this new species.

#### Phenology.

Flowering and fruiting occur from late March to late May.

#### Additional specimens examined.

China. Guangxi: Nanning City, Shanglin County, Xiyan Town, Jianglu Village, Damingshan, from Zuitun to Sanbao, 23°30'50.83"N, 108°28'22.42"E, under broad-leaved forest, alt. 473 m, 31 May 2020, *Y. L. Su et al. 450125200531038LY* (IBK00445401!, IBK00445402!, ZJFC!, ZM!); Nanning City, Shanglin County, Dalan River, Damingshan, alt. 460 m, 16 Oct 2011, *L. Wu & J. C. Yang D3254* (IBK00218552!); Nanning City, Shanglin County, Liangjiang Town, Chaoyang River, Damingshan, alt. 1030 m, 23 May 2011, *L. Wu D2120* (IBK00218553!).

#### Conservation status.

Least Concern (LC). The new species is known from four localities in Damingshan National Nature Reserve of Guangxi. These populations are in protected areas where they are not really threatened but need attention at ordinary times (IUCN 2019).

#### Notes.

*Carexdamingshanica* belongs to sect. Lageniformes in having terminal spikes staminate and nutlets apex contracted into a prominent long cylindrical beak (Dai et al. 2010). It is similar to *C.breviscapa* and *C.rhynchachaenium*, but differs from these two species in the characters of spikes, pistillate glumes, utricles and nutlets. The morphological differences of *C.damingshanica*, *C.breviscapa* and *C.rhynchachaenium* are shown in Table 2.

**Table 2.** Morphological characters distinguishing *Carexdamingshanica* from *C.breviscapa* and *C.rhynchachaenium*.

Characters	*C.damingshanica*	*C.breviscapa*	*C.rhynchachaenium*
1. Spikes	3 or 4, aggregated	Many, 3–5 at each node	3–6
2. Lateral spikes	Pistillate, cylindrical, 4–13 mm long	Pistillate or mostly with male part at apex, narrowly cylindric, 3–4.5 cm long	Pistillate, shortly cylindric, 1–2 cm long
3. Pistillate glume	Ovate,1–1.2 mm long, apex acuminate	Ovate-oblong, 2.5–3 mm long, apex rounded	Oblong-elliptic, apex truncate-rounded, occasionally mucronate
4. Utricle	Narrowly fusiform, obtusely trigonous, 2.5–3 mm long	Rhombic-fusiform, trigonous, 3.5–5 mm long	Lageniform with weak constriction at middle, 5–6.5 mm long
5. Nutlet	Narrowly ovoid, 1.5–1.9 mm long	Rhombic-ovoid, 2.5–3 mm long	Rhombic-ovoid, ca. 4 mm long

As above-mentioned, *Carexbreviscapa* was placed in Tristachya clade, therefore its closed species, *C.damingshanica*, is temporarily placed in the Tristachya clade.

### ﻿3. Carex radicalispicula

Taxon classificationPlantaePoalesCyperaceae

Tang & F.T.Wang ex Y.F.Lu & X.F.Jin sp. nov.

DE7AC8A9-FFAB-5AE1-8F4D-D1A0157D1590

urn:lsid:ipni.org:names:77318325-1

Fig. 4A–G

#### Diagnostic description.

This new species is similar to *Carextruncatirostris* S.W.Su et S.M.Xu, but differs in having staminate spikes clavate, 1.5–2 mm wide, pistillate glumes pale yellow-white, 3–3.2 mm long, acuminate or short-awned at apex, nutlets with 3 angles shallowly constricted at middle.

**Figure 4.**
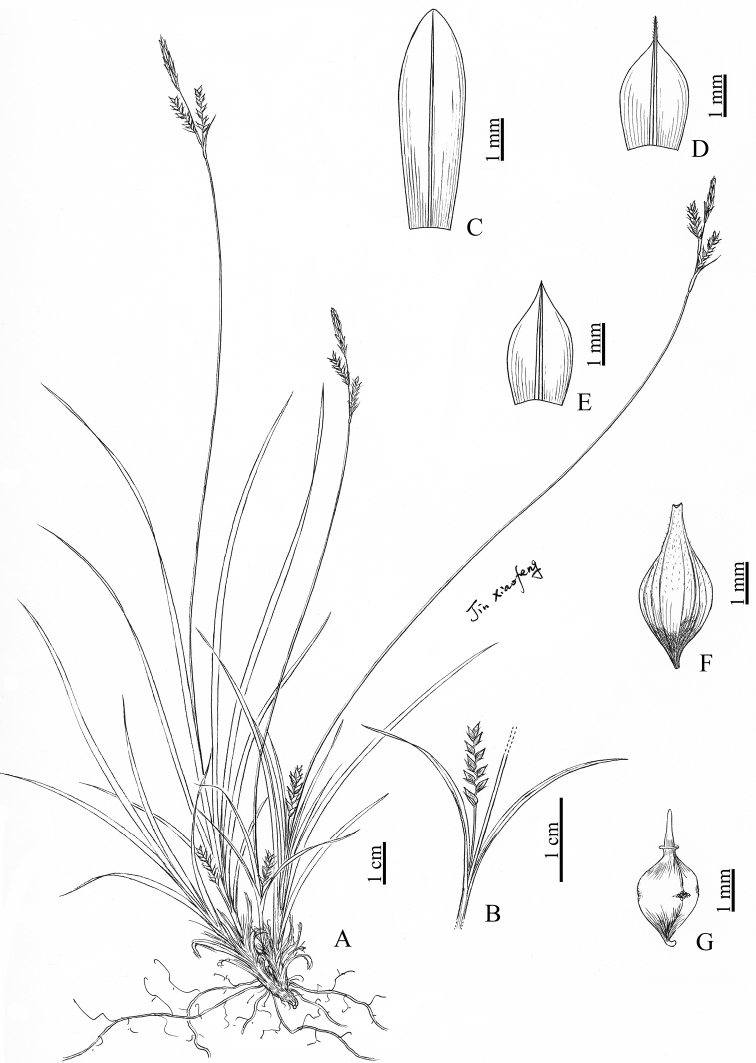
*Carexradicalispicula* sp. nov. **A** habit **B** lowermost spike **C** staminate glume **D, E** pistillate glume **F** utricle **G** nutlet. (Drawn by Xiao-Feng Jin; based on the holotype: *K. L. Chü 6963* in PE).

#### Type.

China. Sichuan: Kangding County, Erdaoqiao, on roadside, alt. 2650 m, 26 May 1940, *K. L. Chü 6963* (holotype: PE!; isotypes: IBSC0654521!, PE!).

#### Description.

Perennial herbs. Rhizomes short, woody. Culms central, loosely turfed, 8–30 cm tall, slender, trigonous, smooth, base with grey-brown sheaths. Leaves shorter than or rarely equal to culms, blades 1.5–2 mm wide, flat, leathery, scabrous on margins. Lowermost bract leaf-like, others setaceous, base with 4–6 mm long sheath. Spikes 3 or 4; terminal spike staminate, clavate, (5–)9–12 mm long, 1.5–2 mm wide; lateral spikes pistillate, with the lowermost arising from the base of culms, shortly cylindrical or cylindrical, 7–15 mm long, 3.5–4 mm wide, 4–12-flowered, with the lowermost peduncle slightly exserted from the sheath. Staminate glumes elliptic-lanceolate, yellow-brown, 4.5–5.5 mm long, apex obtuse, with 1-veined yellow costa. Pistillate glumes broadly ovate, pale yellow-white, 3–3.2 mm long, apex acuminate or with a ca. 0.5 mm long scabrous awn, with 3-veined green costa. Utricles yellow-green, rhombic-ovoid, obtusely trigonous, ca. 3.8 mm long, ca. 1.3 mm wide, longer than pistillate glumes, membranous, obliquely patent, distinctly thinly veined, sparsely pubescent, base gradually cuneate, short-stipitate, apex gradually contracted into a 0.8–1 mm long beak, orifice 2-lobed with short teeth. Nutlets tightly enveloped, pale yellow, rhombic-ovoid, trigonous, ca. 2.5 mm long, with 3 angles shallowly constricted at middle, lateral sides slightly concave above and below, base shortly curved-stipitate, apex abruptly contracted into a discoid-annulate style-base; style base thickened; stigmas 3.

#### Etymology.

The specific epithet ‘*radicalispicula*’ refers to the lowermost spike arising from the base of culm.

#### Phenology.

Flowering and fruiting occur in late May.

#### Conservation status.

Least Concern (LC). The new species was collected by Kuei-Ling Chü (*K. L. Chü 6963*) from the type locality, including two sheets deposited in PE and one in IBSC. The authors carried out a field trip to the type locality in 2019, but failed to locate and collect any similar specimens. The type locality has been disturbed and the quality of the habitat appears to be continuously declining now (IUCN 2019).

#### Notes.

With rhombic-ovoid nutlets abruptly contracted into a discoid-annulate style-base at the apex, 3 angles constricted at the middle, and the lowermost spike arising from a culm base, *Carexradicalispicula* is similar to *C.truncatirostris*. A taxonomic revision of *C.chungii* Z.P.Wang and the allied species has been conducted (Jin 2017), and these very closely related species can be distinguished from each another using the following key.

**Table:** 

1a	Lowermost spikes exserted from the basal sheaths of culms; lowermost bract sheaths < 6 mm long	**2a**
2a	Terminal staminate spikes 1.5–2 mm wide; nutlets shallowly constricted at middle angles; pistillate glumes pale yellow-white, 3–3.2 mm long, acuminate or short-awned at apex	***Carexradicalispicula***
2b	Terminal staminate spikes 0.6–1 mm wide; nutlets constricted at middle angles; pistillate glumes pale brown or yellow-brown, 2–2.5 mm long, emarginate or obtuse at apex	***Carextruncatirostris***
1b	Lowermost spikes exserted from the middle sheaths of culms; lowermost bract sheaths > 1 cm long	**3a**
3a	Terminal spikes 1–3 cm long; pistillate glumes long-awned at apex; staminate glumes short-awned or mucronate at apex	***Carexchungii***
3b	Terminal spikes 3.5–6 cm long; pistillate glumes mucronate at apex; staminate glumes acute at apex	***Carexnanpingensis***

The new species is closed to *Carextruncatirostris*, and the species have nutlets apex abruptly contracted into a discoid-annulate style-base which were divided into two clades in the recent phylogenetic scaffold for the *Carex* classification (Roalson et al. 2021). The species in the Mitrata Clade have shorter lateral spikes and smaller plants, and the new species is temporarily placed in the Mitrata clade.

## Supplementary Material

XML Treatment for Carex fatsuaniana

XML Treatment for Carex damingshanica

XML Treatment for Carex radicalispicula
